# Body Fat Mass, Percent Body Fat, Fat-Free Mass, and Skeletal Muscle Mass Reference Curves for Czech Children Aged 6–11 Years

**DOI:** 10.3390/children8050366

**Published:** 2021-05-04

**Authors:** Vendula Zbořilová, Miroslava Přidalová, Tereza Kaplanová

**Affiliations:** Department of Natural Sciences in Kinanthropology, Faculty of Physical Culture, Palacký University, 771 11 Olomouc, Czech Republic; miroslava.pridalova@upol.cz (M.P.); tereza.kaplanova@upol.cz (T.K.)

**Keywords:** body composition, bioelectrical impedance, centiles, children

## Abstract

Background: Negative lifestyle trends are reflected in overweight and obese children, in which their lack of physical activity results in decreased muscle mass. This study aimed to define age- and sex-specific reference curves for body fat mass (BFM), skeletal muscle mass (SMM), fat-free mass (FFM), and percent body fat (%BF) in Czech children. Methods: Body composition was measured by segmental bioelectrical impedance (BIA, InBody 720). The research sample included 2093 children aged 6–11 years (1008 boys and 1085 girls). Only children whose parents provided informed consent were included. Statistical analysis was performed using SPSS v. 22. The statistical analysis was performed separately by age and sex. Anthropometric data were summarized as means and standard deviation. The percentile curves (P3, P10, P25, P50, P75, P90, and P97) of BFM, FFM, %BF, and SMM were calculated using the gamlss package in R 3.4.2 (R Foundation for Statistical Computing, Vienna, Austria). Results: This study developed age- and gender-specific percentile curves of SMM, FFM, BFM, and %BF for Czech children aged 6–11 years. During childhood, BFM and %BF increased in boys, peaking at approximately 11 years of age. Girls displayed a different pattern of age-related changes in BFM and %BF compared to that in boys. These parameters gradually increased during childhood. This pattern was also observed for SMM and FFM in both sexes. Conclusions: The purpose of this study was to serve as a reference to improve methods to evaluate body composition in Czech children and for comparison with studies worldwide.

## 1. Background

The global increase in the prevalence of overweightness and obesity in all age categories [1,2,3] related to lack of physical activity (PA) and inappropriate nutritional habits [4] has motivated the investigation and rationale for effective education and social and health programs aimed at mitigating these negative trends. The incidence of obesity is increasingly occurring in lower age categories [3,5]. Obesity, a disease associated with increased levels of economic development of a country and characterized by excessive accumulation of fat in the human body, is a risk factor for many non-infectious diseases in adult and childhood populations [6,7]. Furthermore, pediatric obesity tends to persist into adolescence [8] and even into adulthood, with severe health consequences [9,10,11,12]. 

In connection with increasing overweightness, obesity as well as underweight status during childhood negatively affect motoric fitness, which depends on neuromuscular coordination, muscle fitness, and development.

Overweightness and obesity are associated with excess adipose tissue, the quantity of which is measured for the evaluation of body composition. Adipose tissue is the largest endocrine organ in the body; its increase leads to major metabolic changes and, consequently, to somatic transformation in the body [13,14]. Skeletal muscles are also very important, as the ratio between muscle and fat mass is often used to assess metabolic health [15]. Skeletal muscles are the key component of nutritional assessment and are increasingly recognized as an independent indicator of metabolic health [16,17]. 

The most effective treatment for childhood obesity is primary prevention [18]. Central role in the prevention is played by somatic diagnosis. Through somatic diagnosis, we are able to determine and then, within the development, compare the growth channeling of selected somatic parameters of child in percentile charts. Comparison of measured anthropometric data of a child to the population average by utilizing growth charts is one of the basic methods for growth evaluation. Growth charts are an indispensable part of pediatric practice for their ease of use in assessing growth and development [19,20]. Anthropometric surveys carried out at regular intervals are important indicators of children’s health and the most appropriate way to assess the nutritional and health status of the pediatric population [21,22].

The Czech Republic is among the countries with available national growth reference data for body height and weight, body mass index (BMI), and circumference parameters. These data were collected by National Anthropological Surveys performed in 1981, 1991, and 2001 [23]. The Czech Republic ranked among the countries with a tradition of extensive anthropological research, which provided important growth characteristics of the Czech population, both children and adults, which were carried out in connection with the Czechoslovak Spartakiads in 1955, 1960, 1965, 1975 [24], 1980, and 1985 [25,26,27] as well as a number of other major studies [28,29]. Accordingly, it is essential not only to update the national growth references for the aforementioned parameters, but also to create a national growth reference for body composition parameters that have not yet been considered.

Therefore, this study aimed to create age- and sex-specific skeletal muscle mass (SMM), body fat mass (BFM), percent body fat (%BF), and fat-free mass (FFM) percentile curves in Czech children 6–11 years of age.

## 2. Methods

### 2.1. Subjects and Anthropometric Measurements

Primary schools from all regions of the Czech Republic (14 regions) were contacted for the implementation of the research. A total of 60 schools were addressed. A total of 23 schools (approx. 1/3 of all addressed schools) from 5 regions of the Czech Republic (approx. 1/3 of all regions of the Czech Republic) expressed interest in cooperation. The addressed schools were selected randomly or on the basis of previous cooperation in another research. The research was carried out from 2014 to 2018. The research sample included 2093 apparently healthy children aged 6–11 years (1008 boys and 1085 girls). The parents of all children accepted and signed written informed consent.

The children’s body composition was predicted using an InBody 720 device. This device provides information about BFM, %BF, visceral fat, SMM, FFM, total body water, intracellular water, extracellular water, etc. The measurement procedure required the subject to stand in bare feet on the analyzer and to hold a pair of handgrips, one in each hand. The measurements were performed in selected classes in primary schools or school club rooms in the morning under standard conditions while maintaining a high level of hygiene. These conditions refer to the manufacturer’s recommended standard conditions for the InBody 720 device, which works by MF-BIA method. The headteachers, teachers, parents, and guardians were informed in advance of which rules must be followed before measurement. The headteachers received these rules in a letter, where the design of the study was described, as well as its intentions and research methods. The boys and girls were measured separately in their underwear. Data obtained from the InBody 720 device were processed using the Lookin Body 3.0 program. 

### 2.2. Statistical Analysis and Centile Curves

Statistical analysis was performed using IBM SPSS Statistics for Windows, version 22.0 (IBM Corp., New York, NY, USA). The statistical analysis was performed separately by age and sex. Anthropometric data were summarized as means and standard deviation. The percentile curves (P3, P10, P25, P50, P75, P90, and P97) of BFM, FFM, %BF, and SMM were calculated using the gamlss package in R 3.4.2 (R Foundation for Statistical Computing, Vienna, Austria).

## 3. Results

Table 1 shows the means and standard deviations of anthropometric and body composition parameters for each age and sex. There are numerical disproportions between the various age categories. However, they are based directly on the number of children in the individual classes of the first stage of the relevant primary schools.

**Figure 1:**
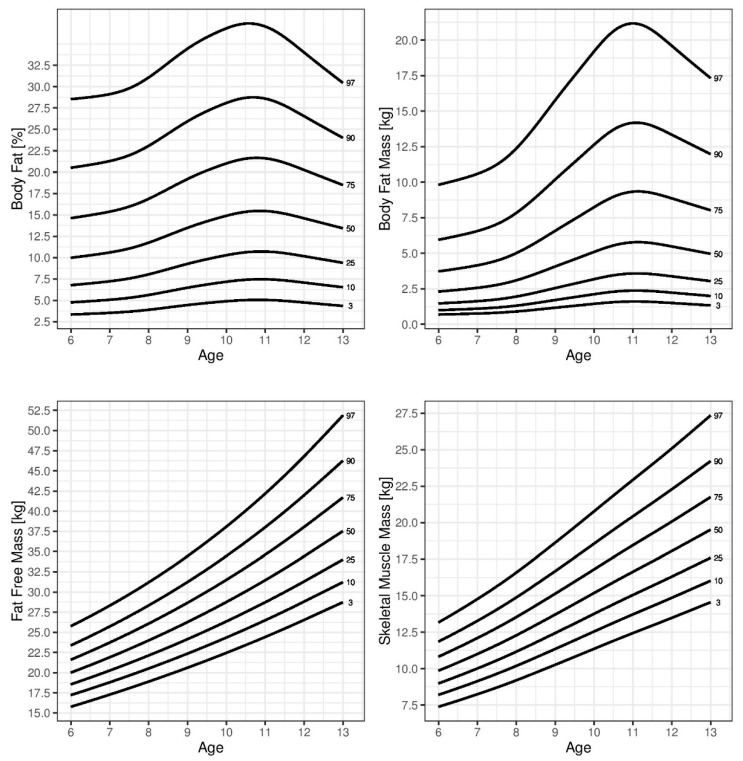
Curves of the 3rd, 10th, 25th, 50th, 75th, 90th, and 95th percentiles for skeletal muscle mass (SMM), body fat mass (BFM), fat-free mass (FFM), and percent body fat (%BF) by age in boys.

**Figure 2:**
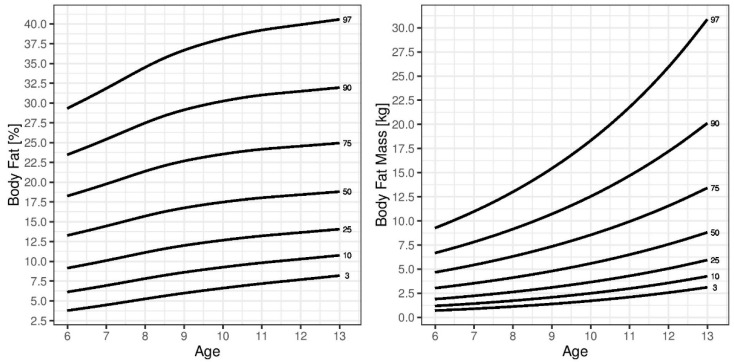

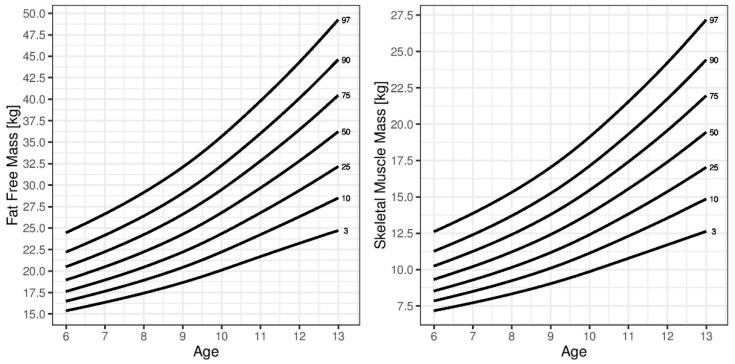
Curves of the 3rd, 10th, 25th, 50th, 75th, 90th, and 95th percentiles for skeletal muscle mass (SMM), body fat mass (BFM), fat-free mass (FFM), and percent body fat (%BF) by age in girls.

In general, SMM was higher in boys, while BFM and %BF were higher in girls. During childhood, BFM and %BF increased in boys, peaking at approximately 11 years of age. Girls displayed a different pattern of age-related changes in BFM and %BF compared to those in boys. These parameters gradually increased during childhood. This pattern was also observed for SMM and FFM in both sexes. The reference percentile curves generated for %BF are illustrated in Figure 1 (boys) and Figure 2 (girls). A sex-based difference was observed in the shape of the fat percentile curves, in which those for the lower percentiles in boys were flatter than those in girls and the %BF showed a greater increase in girls than that in boys around 10 years of age. The median %BF percentile of boys was also lower than that in girls. The reference percentile curves generated for SMM and FFM are also shown in Figure 1 (boys) and Figure 2 (girls). Both sexes showed similar patterns in the shapes of the SMM and FFM percentile curves. Table 2, Table 3, Table 4 and Table 5 show the SMM, FFM, BFM, and %BF values across the percentiles by age and sex.

## 4. Discussion

The results of this study provided age- and sex-specific reference curves for skeletal muscle mass, body fat mass, fat-free mass, and percent body fat measured by multi-frequency bioelectric impedance (MF-BIA) for Czech children aged 6 to 11. 

Currently, pediatric literature often relies on BMI to classify children as overweight and obese. Although BMI is an important epidemiologic and clinical tool [30], it does not distinguish between FFM and BFM; thus, individuals with the same BMI show varying levels of fatness [31]. 

MF-BIA is a method commonly used for body composition analysis. This method offers a simple, non-invasive, and fast way to determine the body composition of test subjects in clinical practice as well as in larger field measurements and is considered to be an adequately valid and reliable method in both adult and childhood populations. Examination of children using this method is fast. Children are not subjected to a long examination and can stand still and calm for the duration of the analysis [32,33,34,35,36]. 

Comparison of our results to those of other studies was challenging due to differences in sample size, body composition analysis, subject ages, procedures, and study design. However, several studies used designs similar to that in the present study. 

Studies that also used the BIA method to generate body fat reference curves included those by Kurtoglu et al. [37] in Turkish of children and adolescents; Papandreou, Malindretos, and Rousso [38] in children from northern Greece; and Escobar-Cardozo et al. [39] in children and adolescents from Bogota, Colombia.

We compared our results to those reported by McCarthy et al. [40] in a study including children from Southern England. The authors reached a similar conclusion to that in the present study, in which the % BF percentile curves during childhood in boys peaked at 11 years. In girls, the percentile curves had continuously increased during childhood. Similar observations were reported in a study providing age- and sex-specific (% BF) reference percentiles for US children and adolescents [41]; however, %BF in this study was derived from skinfold thicknesses. Alpizar et al. also measured BFM using skinfold thickness measurements [42] to develop body composition percentile curves in Mexican children aged 6–12 years. Czech children, in comparison to Mexican children, had significantly lower proportions of body fat in all age categories. In contrast, FFM (kg) was higher in Czech boys in all younger age groups and girls aged 6–9 years. In Czech boys, %BF increased during childhood, peaking at approximately 11 years. The highest increase in %BF in Mexican boys occurred at 9–10 years of age. Both Czech and Mexican girls showed a different pattern of age-related changes in %BF compared to those in boys. The percentile curves of %BF showed smooth courses during childhood.

McCarthy et al. also developed percentile curves for SMM in children [43]. This study measured body composition by segmental bioelectrical impedance, with results consistent with those in the present study. SMM in both boys and girls increased with age. The courses of the percentile curves increased relatively steeply over the respective age spectrum. Czech children, however, had a higher representation of SMM in all age categories. This was also observed when comparing FFM values between Czech and Asian children. These findings are comparable to those reported by Chiplonkar et al. [44] using the bioelectric impedance method and by Liu et al. [45] and Kim, Hong, and Kim [46] using dual-energy X-ray absorptiometry to determine body composition.

To assess the physical development of children, pediatricians in the Czech Republic have at their disposal growth charts of body height, body weight, BMI, and selected circumferential parameters, such as the abdominal circumference. Such an assessment is the easiest way to determine the health and nutritional status of a child. Percentile curves of %BF, body fat mass, skeletal muscle mass, and fat-free mass have not been constructed for this purpose yet. Due to changes in the lifestyle of the population in terms of decreasing physical activity and increasing trend of sedentary behavior, often accompanied by unhealthy nutrition, we believe that it is necessary to assess not only the total body weight of the child, but also to check the proportion of the individual body fractions. 

## 5. Limitations

The limitations of this study include the fact that MF-BIA is not a gold-standard method for body composition analysis. The choice of participating primary schools depended on their headmasters’ willingness to cooperate. From a biological point of view, the limits of the study include the hydration of FFM with respect to age and maturation.

## 6. Conclusions

This study provided age- and sex-specific SMM, BFM, %BF, and FFM percentile curves in Czech children aged 6–11 years. We proposed the use of the BFM and %BF percentile curves presented here as an alternative or addition to BMI curves. FFM and SMM percentile curves can contribute to a comprehensive assessment of body composition and inform the growth trends of Czech children. The purpose of this study was to provide a national reference to enrich the methods for evaluating overall body composition in early school-aged Czech children. Due to the fact that in recent decades there have been significant somatic changes in early school-aged children in terms of their acceleration in the context of increasing overweightness and obesity, worldwide, it is necessary to monitor not only BMI, but also the amount of individual body fractions. Acceleration of biological development may obscure the higher prevalence of overweight and obesity as well as latent obesity in the pediatric population. Specifications of individual body fractions in the form of percentile curves can serve as a tool to assess latent obesity and adequate development of muscle mass in children. The created percentile curves could be suitable supplements for Czech pediatricians for assessing the health and nutritional status of a child. 

## Figures and Tables

**Table 1:** Anthropometric and body composition characteristics.

Age (Years)	n	Height (cm)	Weight (kg)	SMM (kg)	FFM (kg)	%BF	BFM (kg)
Boys							
6	74	125.5 ± 6.1	24.9 ± 4.5	10.9 ± 1.7	21.7 ± 2.8	12.2 ± 6.3	3.3 ± 2.3
7	223	132.1 ± 6.3	29.2 ± 5.4	11.7 ± 1.9	23.0 ± 3.1	12.3 ± 7.0	3.6 ± 2.9
8	254	134.2 ± 5.7	30.6 ± 6.5	13.3 ± 2.1	25.8 ± 3.4	14.5 ± 8.3	4.9 ± 4.0
9	179	139.0 ± 6.7	33.5 ± 7.0	14.5 ± 2.3	27.8 ± 3.9	15.7 ± 8.0	5.7 ± 4.2
10	148	145.4 ± 7.5	38.0 ± 9.3	16.3 ± 3.0	30.9 ± 5.0	17.2 ± 8.2	7.1 ± 5.3
11	130	148.3 ± 7.1	40.2 ± 8.4	17.5 ± 2.7	32.8 ± 4.6	17.0 ± 8.7	7.4 ± 5.3
Girls							
6	95	124.0 ± 6.8	24.8 ± 6.1	10.3 ± 2.0	20.7 ± 3.4	15.4 ± 7.5	4.1 ± 3.1
7	255	127.5 ± 6.4	26.0 ± 5.1	10.8 ± 1.7	21.5 ± 2.9	16.0 ± 7.9	4.5 ± 3.1
8	251	132.1 ± 6.9	29.2 ± 5.7	12.0 ± 1.9	23.6 ± 3.3	18.2 ± 8.2	5.6 ± 3.6
9	174	136.9 ± 7.0	31.6 ± 7.5	13.1 ± 2.3	25.4 ± 3.9	18.0 ± 8.6	6.2 ± 4.6
10	177	144.6 ± 7.9	36.7 ± 8.6	15.3 ± 2.9	29.1 ± 4.8	19.1 ± 8.3	7.5 ± 4.9
11	133	147.0 ± 7.4	39.0 ± 9.6	16.2 ± 2.9	30.8 ± 4.9	19.5 ± 8.6	8.2 ± 6.0

Note: Mean ± standard error. SMM (kg), skeletal muscle mass; BFM (kg), body fat mass; BF (%), percent body fat; FFM (kg), fat-free mass.

**Table 2:** Tabulated skeletal muscle mass (SMM) centile values by age.

Percentile							
Age (Years)	3	10	25	50	75	90	97
**Girls**							
6	7.2	7.8	8.5	9.3	10.2	11.3	12.6
7	7.7	8.5	9.3	10.2	11.3	12.4	13.9
8	8.3	9.2	10.1	11.2	12.4	13.7	15.3
9	9.0	10.1	11.2	12.4	13.8	15.2	17.0
10	9.9	11.1	12.4	13.9	15.5	17.1	19.1
11	10.8	12.3	13.8	15.5	17.4	19.3	21.5
**Boys**							
6	7.4	8.2	9.0	9.9	10.8	11.8	13.2
7	8.2	9.1	10.0	11.0	12.1	13.3	14.8
8	9.2	10.2	11.2	12.3	13.5	14.9	16.6
9	10.3	11.3	12.4	13.7	15.1	16.7	18.6
10	11.4	12.5	13.8	15.2	16.8	18.6	20.8
11	12.4	13.7	15.0	16.6	18.4	20.4	22.9

**Table 3:** Tabulated body fat mass (BFM) centile values by age.

Percentile							
Age (years)	3	10	25	50	75	90	97
**Girls**							
6	0.7	1.2	1.9	3.0	4.7	6.7	9.3
7	0.9	1.4	2.2	3.5	5.4	7.8	11.0
8	1.1	1.7	2.6	4.1	6.3	9.1	13.0
9	1.4	2.1	3.1	4.8	7.3	10.7	15.4
10	1.7	2.5	3.7	5.6	8.5	12.5	18.3
11	2.1	3.0	4.3	6.5	9.9	14.7	21.8
**Boys**							
6	0.7	1.0	1.5	2.3	3.7	6.0	9.8
7	0.8	1.1	1.6	2.6	4.2	6.6	10.6
8	0.9	1.3	2.0	3.1	5.0	7.8	12.4
9	1.2	1.7	2.6	4.1	6.6	10.2	15.8
10	1.4	2.1	3.2	5.1	8.2	12.6	19.2
11	1.6	2.4	3.6	5.8	9.3	14.2	21.2

**Table 4:** Tabulated fat-free mass (FFM) centile values by age.

Percentile							
Age (Years)	3	10	25	50	75	90	97
**Girls**							
6	15.4	16.5	17.6	19.0	20.5	22.2	24.5
7	16.4	17.6	19.0	20.5	22.2	24.2	26.7
8	17.4	18.9	20.5	22.2	24.2	26.4	29.1
9	18.7	20.4	22.2	24.3	26.6	29.1	32.1
10	20.1	22.2	24.3	26.8	29.5	32.3	35.7
11	21.7	24.2	26.8	29.7	32.8	36.0	39.8
**Boys**							
6	15.8	17.2	18.5	20.0	21.6	23.4	25.8
7	17.3	18.8	20.3	21.9	23.7	25.7	28.3
8	18.9	20.5	22.1	24.0	26.1	28.3	31.2
9	20.6	22.4	24.2	26.3	28.7	31.2	34.4
10	22.4	24.3	26.3	28.8	31.5	34.4	38.1
11	24.4	26.5	28.7	31.5	34.6	38.0	42.2

**Table 5:** Tabulated percentages of body fat (%BF) centile values by age.

Percentile							
Age (Years)	3	10	25	50	75	90	97
**Girls**							
6	3.8	6.1	9.1	13.3	18.2	23.5	29.3
7	4.5	6.9	10.1	14.5	19.8	25.4	31.8
8	5.2	7.8	11.1	15.7	21.4	27.5	34.5
9	6.0	8.6	12.0	16.7	22.7	29.1	36.7
10	6.6	9.3	12.7	17.5	23.5	30.2	38.2
11	7.2	9.8	13.2	18.0	24.2	31.0	39.2
**Boys**							
6	3.3	4.7	6.8	10.0	14.6	20.5	28.5
7	3.5	5.1	7.2	10.6	15.4	21.3	29.1
8	3.9	5.6	8.0	11.7	16.9	23.1	31.1
9	4.4	6.5	9.3	13.5	19.2	25.9	34.4
10	4.9	7.2	10.3	14.9	21.0	28.1	36.8
11	5.0	7.5	10.7	15.4	21.6	28.6	37.1

## Data Availability

All data generated or analyzed during this study are included in this published article. The datasets used and/or analyzed during the current study are available from the corresponding author on reasonable request.

## Abbreviations

BFM	body fat mass
%BF	percent body fat
SMM	skeletal muscle mass
FFM	fat-free mass
BMI	body mass index
MF-BIA	multifrequency bioelectrical impedance
PA	physical activity
